# International perspectives on implementation of system change in family mental health

**DOI:** 10.3389/fpsyt.2026.1705868

**Published:** 2026-02-24

**Authors:** Melinda Goodyear, Becca Allchin, Bjørg Eva Skogøy, Anne Grant, Kristin Stavnes, Bente Weimand, Sophie Isobel, Kathleen Biebel, Joanne Nicholson, Scott Yates, Rochelle Hine, Lina Gatsou, Bitta Söderblom, Karin van Doesum, Adrian Falkov, Ron Shor, Juliet Collins, Clemens Hosman, Stella Laletas

**Affiliations:** 1 School of Rural Health, Faculty of Medicine, Nursing and Health Sciences, Monash University, Clayton, VIC, Australia; 2 Emerging Minds, National Workforce Centre for Child Mental Health, North Melbourne, VIC, Australia; 3 Eastern Health Mental Health Program, Eastern Health, Melbourne, VIC, Australia; 4 Nordland Research Institute, Bodø, Norway; 5 School of Nursing and Midwifery, Queens University Belfast, Belfast, Northern Ireland, United Kingdom; 6 The Regional Centre for Eating Disorders (RESSP), Nordland Hospital Trust, Bodø, Norway; 7 Division Mental Health, Department of Research and Development, Akershus University Hospital, Lorenskog, Norway; 8 Faculty of Health and Social Sciences, Center for mental health and substance use, University of South-Eastern Norway, Drammen, Norway; 9 Sydney Nursing School, Faculty of Medicine and Health, University of Sydney, Sydney, NSW, Australia; 10 MassAbility, Boston, MA, United States; 11 Institute for Behavioral Health, The Heller School, Brandeis University, Waltham, MA, United States; 12 School of Applied Social Sciences, De Montfort University, Leicester, United Kingdom; 13 Institute of Health, Health Policy and Social Care Research, DeMontford University, Leicester, United Kingdom; 14 Child and Adolescent Mental Health Services, Leicestershire Partnership National Health Service Trust, Leicester, United Kingdom; 15 Faculty of Social Sciences, University of Helsinki, Helsinki, Finland; 16 Radboud University, Nijmegen, Netherlands; 17 Impluz, Dimence groep, Deventer, Netherlands; 18 The Willoughby Clinic, Willoughby, NSW, Australia; 19 Paul Baerwald School of Social Work and Social Welfare, The Hebrew University of Jerusalem, Jerusalem, Israel; 20 School of Educational Psychology and Counselling, Faculty of Education, Monash University, Melbourne, VIC, Australia

**Keywords:** child mental health, delphi study, family focused practice, implementation science (MeSH), parenting (MeSH), system change, mental disorders, workforce development

## Abstract

**Introduction:**

Parental mental illness is a major public health issue across the globe with well-known intergenerational impacts on children. There is a wide body of evidence supporting the effectiveness of a range of interventions supporting families, however, their implementation has rarely been sustained across public health systems. Systemic change is an important part of workforce development and known to be crucial to embed and sustain practice, policy and structural initiatives in services for families. While much is known about the barriers to implementing family focused approaches within organizations and systems, less is known about how systems change occurs and what supports systems change to improve outcomes for families.

**Methods:**

This study uses a Delphi method, with 103 experts from 17 countries participating to identify systems change factors from their own experience and to build consensus about key strategies required across the globe to support systems change in health, education, social welfare and mental health services.

**Results:**

The findings identify that systems change can be defined as any workforce, policy, legislation or other mental health promotion strategy that collectively contributes to improving outcomes for parents with mental illness, their children and their families. A systems approach to improve outcomes for families where a parent has a mental illness requires partnerships and collaboration between services and sectors affecting families (mental health, welfare, primary health, education, social care, public health), social and health policy development, and families themselves. Success in system change requires a focus on change at all levels of the system for momentum building, leadership support, the use of relevant data and reporting mechanisms, establishing practice competency and collaborative care, and being able to reflect and adapt to changing conditions and structural barriers.

**Discussion:**

A focus on system change for supporting families where a parent has a mental illness appears to require the combination of many strategies and factors, with international approaches to knowledge sharing imperative to support implementing, resourcing and sustaining change.

## Introduction

Parental mental illness is a major public health issue internationally with well-known intergenerational effects (1). It is estimated that 23% of children worldwide have a parent with a mental illness (2). People with mental ill health and their families are more likely to also be disadvantaged by other social determinants of health such as poorer access to housing, education, healthcare, social and community connection and a living income (3–5). Without appropriate supports in place, the impacts for the whole family can be complex and long lasting, influencing the mental health and wellbeing of family members across the generations (6, 7).

Family focused practice (FFP) has been shown across the United States, United Kingdom, Scandinavia and Australasia to support families to build their resilience and improve wellbeing outcomes for parents and their children when there is mental illness (8–10). There is now compelling international evidence demonstrating the effectiveness of a range of interventions in improving outcomes for children of parents with mental illness in particular (8, 9). Those interventions with a strong evidence base include, but are not limited to, Family Talk, Let’s Talk About Children, Child Talks+, CHAMPS peer support groups, the Family Model and more recently the “It Takes a Village” Approach (8, 11–18).

There has been a growing awareness of the importance of implementation on the integration of FFP that support families when a parent has a mental illness seen in efforts in Australia, Austria, Finland, Northern Ireland, Norway, UK, USA, Japan, China and Thailand as too often, promising interventions fail to be sustained over time. These have sought to provide insight into factors that influence the successful implementation of these evidence-based programs into routine practice (19–22). Implementation efforts may be targeted at systems of care across multiple settings and sectors that cater to the needs of families where a parent has a mental illness. Common child and adult focused settings and sectors include, but are not limited to: Mental Health, Child and Family Services, Child Protection, Community Service Organizations, Consumer and Carer Advocates, Police, Aboriginal Family Services & Maternity Services (23). Some implementation efforts have narrowed their focus to an individual sector such as primary health (24) whilst others have taken a wider focus across multiple sectors (25, 26). Several of these studies draw on implementation theories such as the Consolidated Framework for Implementation Research (CFIR), and highlight the need to examine the multiple influences that can affect sustained implementation of programs (27). These includes barriers and facilitators from the outer setting, the context of the intervention and even the implementation process as it is planned and delivered. These multi-component strategies are also further described as interacting to produce the change in the environment that helps to embed and sustain changes in practice (28).

Research has also focused on examining ways to address barriers to FFP and influence change at different levels within systems that can offer support to families, including at the organizational (29) or practitioner level (30); and/or change across multiple levels including government/legislative (25) influencing the broad policy, political, cultural and fiscal contexts nationally. The localized nature of most funded initiatives limits the capacity for research studies to be designed with a whole system view that examines the impact of workforce development and system change initiatives simultaneously. Additionally, while sustained use of FFP in real world settings has been limited by known barriers (31), there is less known about ‘what has worked’ to change systems of care supporting families where a parent has a mental illness, and how the architects of these few successes have managed to overcome these barriers.

Identifying the barriers to change and how they interact has contributed to the realization that multiple drivers of the desired practice change are required at multiple leverage points within an organizational or system structure (32–34). Past research highlights the importance of creating favorable policy and funding environments that can influence organizations and the services they offer, and that having no formal mandate of this type to implement FFP is a major barrier in the field (35–38). On the other hand, research across multiple countries also shows that legislative changes to policy at government level in isolation, have failed to translate to a significant change at the service/practitioner level (20, 25, 39, 40). This work also draws upon an understanding of system change, a fundamental shift in the working of a system including its structure, roles, culture and/or function to improve its collective functioning as a whole to improve outcomes (41).

At the system level, research shows that in order to improve outcomes for children and their families, there is a need to change the way multiple service systems support them to include early identification and early access to evidence-based practices that can help prevent or reduce the negative impact of issues they are facing (10, 42, 43). However many studies demonstrate that the systems of care that these families come into contact with are under-resourced and struggling to deliver a responsive early intervention approach that implements evidence-based practice (42, 44, 45). Implementation and sustainability of these evidence based practices across the continuum of care has been met with considerable challenges internationally (20, 35, 37, 46, 47). Whilst there is evidence of multiple efforts worldwide that aim to change these systems, much of the research in this area focuses on the implementation, but not sustainability, of a specific FFP intervention, model or program (29).

Implementation studies of family interventions targeting parental mental illness show that a lack of whole organization engagement and of coordination of the multiple factors that support change can hinder the implementation of FFP (48, 49). Significant barriers arise from the influence of conflicting paradigms within sectors, siloed systems and attempting to implement holistic and family-centered approaches within settings that are driven by individual focused bio-medical models. While many of the studies in the field of parental mental illness give insight into the multiple factors involved in the systems and what factors may facilitate or prevent change, they do not tend to take a systems level approach. In taking a system change approach, there is a need to focus on the components of a system and the interactions between them that can be manipulated to transform that system to ensure better outcomes for its beneficiaries (50). In the case of supporting early intervention for child mental health, a system approach draws together numerous service systems such as adult and child mental health services, child protection and social care, and primary health, education and community-based services that might be addressing risk and protective factors for child mental health, a significant factor being parental mental health status (20). These service systems often interact (but rarely collaborate) to influence outcomes for children experiencing mental health risk factors and can form part of a stepped care approach to promoting child mental health (51).

Implementation science as a field has typically concentrated on more ‘push’ approaches to seeking change, where an evidence-based model of practice is drawn from outside the context and ‘pushed’ into the ‘real world setting’ with the aid of an implementation support model (52). However, when exploring the change strategies used in real-world settings, more participatory and collaborative approaches have been associated with success in seeking a change in systems of care for families (52, 53). This has led to a call for greater partnerships between intervention developers, implementation researchers and practice settings to support more iterative learning to lessen the research-practice gap (52, 53). Implementation researchers highlight that these findings indicate the need to create a ‘stronger connection between implementation research and [actual] implementation practice (52).

Few studies have sought to ascertain the experiences of those ‘real world’ change agents and influencers at the system change level for families experiencing mental ill health. This is particularly important given the dearth of literature on successful sustainment strategies (31, 54) and the lack of use of implementation support models for change in practice (52). Previous research conducted by a systems-change sub-group of an international research collaborative for change in parent and child mental health (https://www.parentandchildmentalhealth.org/) set the foundations for this current study. Their findings proposed a systems approach showcasing international examples of implementation success and advocating for deeper understanding of strategies used across those countries where initiatives have developed (20). The present study continues the work of the international collaborative exploring common system change strategies utilized in systems of care for these families from the perspectives of ‘experts’ across the world engaged in the change journey. Representation was sought from 17 countries connected to system change initiatives for families where a parent experiences mental ill health across North America, Europe, Australasia and the Middle East to understand the contextualized factors that were deemed important for system change to support families where a parent has a mental illness in their respective country.

## Materials and methods

### Study design

A two-round Delphi consensus method was employed to identify system change factors deemed important from key informants across the world. A Delphi study design uses several cycles of data collection, analysis and feedback to build consensus from a group of experts that preserves their anonymity and mitigates the effects of ‘group think’ and powerful individuals influencing dynamics and outcomes (55). This iterative method utilized an initial qualitative data collection method, followed by presentation of themes for feedback through quantitative ranking by participants, collected in the form of sequential online individual surveys via the survey platform Qualtrics (56).

The aim of this study was to understand key strategies globally that are associated with system change in health, education, social welfare and mental health services to support families where a parent experiences mental ill health. In the first round, open ended questions were developed by the systems-change sub-group of the international research collaborative, and key informants were asked to explain the developmental journey of system change in their respective country (through an online survey). Participants were asked to reflect on systems change that had increased support for families where a parent experiences mental ill health and identify strategies and initiatives that were either implemented locally, regionally, and/or nationally to drive a change to the way care was provided to children and/or their families. The round one online survey was open over a 12-month period in 2017 via the Qualtrics survey platform. This data was then coded by the same group of researchers using the framework method (57), which systematically processes large bodies of data utilizing a matrix (see analysis section for more details), and leads to an overarching analytic framework and subsequent development of groupings representing each of the answers to the Delphi questions. Through this framework, significant strategies and barriers were then fed back to key informants in round two via a quantitative questionnaire, to rank and rate the relevance and importance of each driver identified. The round two survey was open for 10 months across 2019–2020 in the form of an online survey via Qualtrics.

### Research questions

Through this Delphi process, we sought consensus on the following research questions:

1. What are the strategies or initiatives used across the world to advance systems change in FFP?
2. What are the core elements and level of importance of various successful and unsuccessful initiatives to system change approaches?

### Ethical considerations

Ethics approval was obtained from Monash University Human Research Ethics Committee by the lead researcher (MG), and then approved by local governance of the co-investigators’ affiliated countries where required. Several countries required administrative approval processes for research to be undertaken within their context. Informed consent was obtained electronically through an initial sign-up page on each of the round one and round two surveys. Participants were asked to view the explanatory statement regarding their participation in the project and then respond (tick the box) to indicate their willingness to consent.

### Recruitment and inclusion criteria

Key informant ‘experts’ were classified as service development workers, policy makers, managers, practitioners, and health or welfare workers who had expertise at working at the system level as well as an understanding of change over time in their respective countries of work. The inclusion criteria included psychologists, social workers, occupational therapists, mental health nurses, psychiatrists, researchers, consumer advocates, government officials, policy makers, and administrators.

For the purposes of the study, systems change was defined as ‘any workforce, policy, legislation, or other mental health promotion strategy or development that aimed to identify and support parents with mental illness and their families, including their children’. This was a working definition developed by the system change group of researchers who were part of the Prato International Research Collective (Prato Collaborative), and drew on earlier published research into systems change across countries by the research group (20).

In round one, experts were identified through the Prato Collaborative as part of the systems change research group’s networks and professional affiliates in their respective countries (20). Invitations were sent to key change makers within each country and known to the system change research group. Respondents were asked to participate in the study if they identified as working at the system change level within their respective countries, as outlined above. These individuals were invited to participate in the study via email and encouraged to pass on the invitation to participate to other relevant informants in their network in their respective country based on the above criteria regarding identification as a system change maker.

Participants from round one who had indicated a willingness to be recontacted, were emailed by the research team and invited to complete the second round of the Delphi study via a link to the questionnaire. Participants and the research team were also asked to forward the questionnaire link to other participants who might have met the inclusion criteria from round one, but not participated at the time. These two rounds of the Delphi study were conducted over a three-year period prior to and during the beginning stages of the COVID-19 pandemic. A secondary narrative analysis of the round one data was conducted following the round one data analysis and is reported elsewhere (58).

### Data collection and analysis

#### Round one

The first round of the Delphi study used a questionnaire developed by the systems change working group of the international research collaboration for parent and child mental health which included demographic questions and a series of open-ended questions. The demographic questions included items about the participant’s demographics (age, gender, country, professional background, years of experience in profession, current professional role, type of organization). A series of open-ended questions related to system change and FFP in their country formed the bulk of the questionnaire.

The open-ended questions asked them to describe from their perspective:

- steps or approaches undertaken within their country to seek system change, with examples locally, regionally and or nationally;
- what has worked (or not) to facilitate mental health and welfare service system change to support early intervention and prevention of mental illness and promote family mental health,
- what most significant change/s had occurred to achieve a shift in a mental health and welfare service system(s) to address the care of families where a parent has a mental illness?

Participants were asked to draw from their experiences or observations from their own lived experience of change. Both the questionnaires and responses were translated for participants from countries where English was not the official language, by a researcher in the working group representing that country, and responses were back translated to English for analysis. The ‘most significant change’ question was included as it can be a helpful way to identify key factors from personal accounts of change when dealing with the challenges of analyzing complex systems and programs (59).

In preparation for the second round of the Delphi Study, a framework analysis was undertaken for open-ended responses to identify key strategies based on the informant’s individual perspective and experience with systems change in each country (57, 60)). The analysis proceeded through successive steps of familiarization with the data; coding and developing a framework [or matrix]; charting the data and mapping and interpreting (57, 60). Framework analysis is a flexible approach to analyzing qualitative data such as those collected in the open-ended Delphi questions. It enables the production of structured outputs of qualitative data in a clear framework or matrix, and is particularly useful for dealing with large datasets in multi-disciplinary research (57). Framework analysis was used as it can be helpful to address the contextual and evaluative type research questions that this study was undertaking (61). The multi-stage analysis process involved a small research subgroup (MG, SL, BG, SI, BA, JN) i) reading the responses in full, ii) individually line-by-line coding of responses to each question, iii) meeting to draw together each of our initial codes into an analytical framework across the main categories and themes relating to key strategies and barriers to change. The data was then iv) collated using the analytical framework, before the group met to v) map and discuss all responses to reach a consensus on categories and themes. The main categories of the analytical framework included:

- What is system change,
- System change initiatives - What has worked- What has been done in approaches in their respective country of origin to systems change initiatives that have influenced the care and formal support provided to families where a parent has a mental illness,
- Barriers to change or What didn’t work or got in the way from their experience to affect system change in systems of care for families, and
- The most significant change.

The analytical framework incorporating key strategies and barriers were then presented in a virtual workshop to the larger research group of the Prato collaborative for feedback. Definitions of system change were drafted and the key strategies for system change were drawn from the analytical framework and presented to the research group as statements of key strategies and barriers, to seek agreement to be used as rating statements in the second round of the Delphi study (see Tables for these statements in the results section).

#### Round two

As mentioned, round two’s quantitative questionnaire was developed from the key strategies and barriers in the analytical framework identified in round one. The questionnaire was developed and presented using the Qualtrics digital platform (Qualtrics, Provo, UT) and had three parts; demographics, definitions and ranking statements related to systems change strategies and barriers.

The demographic items included the same items used in round one with an additional item asking if they had participated in round one. In the definitions section, a two-part definition of system change drawn from the round one data, was presented to informants and they were asked to rate their level of agreement with the definition statements. Informants also had the option of making changes or comments to the definitions for further refinement.

In the ranking section of the questionnaire, statements drawn from round one’s key strategies were presented in the form of broad groupings of key strategies and barriers for informants to rank, identify relevance and/or rate for importance in their setting (see Tables for these statements in the results section). These groupings included:

- the importance of the ‘what worked’ statements from their observed experience including key strategies of an initiative that resulted in a change in service delivery models,
- the importance of activities used in their setting at the beginning of their system change journey (*identify and rate)*, and for the sustainment of practice change (*identify only*),
- the importance of ‘barriers to change’ statements from their observed experience. Ranking scales were analyzed on a 0–10 or 0–100 analogue scale, from low to high importance/agreement.

Round two analysis utilized frequencies and mean ranking to identify the degree of consensus for statements and comparing differences between what was important at the beginning and for sustainment. The comments related to the definitional questions were tabled and discussed by the analysis group of researchers for their implications and how they could be addressed. These results were then fed back to the larger research group for discussion. The definitions were further refined in response to the implications that were generated from the group discussion.

### Establishing consensus

While consensus is a requirement of any Delphi study, there is no single definition of consensus in the literature and levels can range from between 51%-100% depending on the aims, findings and characteristics of the Delphi study (55, 56, 62–64). For the purpose of this study consensus was explored at various points with the agreed consensus level was set at 70%, this being mid-range and considered sufficient to indicate a convergence of opinion. In the definitional statement on system change consensus was on agreement with the statements. In the ranking of importance of enablers, barriers and strategies, the focus was on consensus of what was understood to be important. Divergent opinions are also important and so are noted and highlighted within the results.

## Results

### Participant demographics

There were 91 of the 103 respondents in round one that completed the survey representing 17 countries. In round two, 15 out of 18 respondents from nine countries completed the survey. Four of these indicated they hadn’t participated in the previous round, seven indicated they had and four did not identify. In round two, more than half came from four countries: Australia and Northern Ireland (20% each), England and Denmark (13% each).

In both rounds of the Delphi, the majority of respondents were female, over 50yrs old (Round 1:53%; Round 2:60%) and had more than 10 years in their profession (Round 1:84%; Round 2:93%) signifying extensive experience. Most currently worked in mental health services or a university and had a social work or nursing background. See **Supplementary Table S1** for the full participant demographics.

### Findings from the Delphi ratings

Definitions and statements generated from the analysis in round one of the Delphi were ranked and rated for importance by the second round of participants to reach a consensus on what was important for systems change to support families where a parent has a mental illness.

### Consensus on definitions

In round two of the Delphi, a definition generated from round one responses was formed and tested for consensus.

#### Definition of system change in the field

Definition 1 - *Systems change in this field is defined as any workforce, policy, legislation or other mental health promotion strategy to identify and support parents with mental illness and their families, including their children.*

This definitional statement showed a mean agreement level of 8.00 out of a possible 10 (80%) amongst the respondents. Even though we had a high level of consensus, a number of comments were made by respondents that suggested some minor editing of the final definition statement to improve clarity. **Supplementary Table S2** contains the additional comments that were made by respondents and the corresponding minor changes to the definition.

These comments led to the development of an updated definition of systems change:

New Definition 1.1 - *Systems change in this field is defined as any workforce, policy, legislation or other mental health promotion strategy that collectively contributes to improving outcomes for parents with mental illness, their children and their families.*

#### Definition of system change

The next definition involved reaching a consensus regarding possible sectors and the need for partnerships in system change initiatives.

Definition 2 - A *systems approach requires partnerships beyond mental health, and to include sectors like education, welfare, primary health, social care, public health and social policy development that connect with families where a parent has a mental illness.*

While the statement had a high level of agreement (mean agreement of 9.42 out of a possible 10), the definition was edited for clarity following some of the respondents’ comments of the definition. Their feedback and the responding changes to the definition of what system change requires is included in **Supplementary Table S3**.

Based on the comments, the definition was refined to the following:

New Definition 2.1 - A *systems approach to improve outcomes for families where a parent has a mental illness requires partnerships and collaborations between services and sectors affecting families (mental health, welfare, primary health, education, social care, public health), social and health policy development, and families themselves.*

#### What worked to change systems

Following the definitional feedback, the second round Delphi survey asked respondents to rate the importance of identified strategies concerning *what has worked* to change systems. Respondents rated the importance of these strategies (between 0-10) as seen in **Table 1**.

**Table 1:** Rating of strategies for ‘What has worked from your observations of systems change initiatives to support families where a parent has a mental illness?’.

Ratings of the level of importance for what worked to change systems (‘0’ being of no importance and ‘10’ being of most importance)	Mean level of importance (range)
Establishing mechanisms for leadership within organizations to monitor and evaluate the practice change	9.20 (4-10)
Developing practice pathways that result in the collaborative practice across sectors (mental health and social care/welfare for example)	9.00 (5-10)
Establishing mechanisms for leadership within organizations to endorse and authorize the practice change	8.93 (3-10)
Promoting awareness the needs and service gaps for families and children to policy makers and legislators	8.87 (4-10)
Securing dedicated funding to implement workforce development initiatives	8.80 (3-10)
Supporting specific leaders or champions to drive the momentum for change within the organization	8.73 (6-10)
Aligning practice change to philosophical underpinnings of service delivery (i.e. holistic, family centered etc.)	8.67 (4-10)
Implementing evidence-based programs/resources that meet the needs of these families	8.60 (5-10)
Creating reporting and feedback mechanisms that monitor the desired practice change	8.53 (4-10)
Designing or tailoring evidence-based programs/resources that meet the needs of these families	8.47 (5-10)
Being ready with a response to contribute to opportunistic reviews of practice approaches within the targeted service setting	8.47 (5-10)
Creating or facilitating legislation and policy directives that mandate a change in practice for organizations and workforce areas	8.33 (4-10)
Motivating individual practitioners or workers of the importance of the desired practice change	8.20 (3-10)
Promoting awareness of the needs and service gaps for families and children to organizations	8.13 (3-10)
Linking the strategy or workforce development initiative to existing reforms or service development project in similar areas	8.13 (1-10)
Developing practice guidelines to create expectations of the practice change for the workforce	7.80 (1-10)
Continuing to focus on small shifts in practice to promote workforce development that create momentum for change and have a cumulative impact over time	7.73 (5-10)

A high degree of consensus on what was important was found for many of the strategies identified from round one, in terms of what worked to change systems of care. Some degree of difference was found in the level of importance though across different strategies. The highest ranked was for the multi-driver approach of having ‘leadership engaged in using data to monitor practice change’. Also highly ranked was the more complex systemic approach of ‘establishing pathways for collaborative practice across service sectors’ and in creating ‘leadership endorsement’ and engaging ‘policymakers’, ‘legislators’ and creating ‘workforce development initiatives’.

Lesser endorsed strategies (although still highly rated) were strategies that were typically standalone and less likely to be integrated or operating in unison such as the ‘development of practice guidelines’ or a focus on selective areas within an organisation such as ‘motivating individual practitioners’. These were rated by informants as some of the lowest areas of importance. Also interestingly, ‘implementing evidence-based family approaches’ was rated low as well as ‘creating legislation or policy directives’ in terms of importance for what worked to change systems.

#### Barriers to change systems

Next respondents were asked to rate the importance of barriers to change that they had experienced. The level of importance of the barriers are indicated in **Table 2** below.

**Table 2:** Rating of barriers to system change.

Level of importance of barriers to system change in your journey ('0' being of no importance and '100' being of most importance)	Mean level of importance (Range)
Organisational structures that prevent practice development	89.17 (50-100)
Working in silos; a lack of collaborative practice	88.46 (50-100)
Lack focus to sustain practices and progress towards system change	83.85 (50-100)
A lack of focus on implementation supports that lead to change	83.08 (10-100)
Impact of the inner setting of an organisation e.g. workplace culture	82.31 (70-100)
Lack of awareness of social determinants of health leading to detrimental impacts on children of parents with a mental illness	80.83 (20-100)
Impact of the outer setting including sector reform, funding changes, legislation	80.77 (60-100)
Individualized funding and service models that impede a family focus	80.00 (10-100)
Stigma limiting access of support services	77.69 (50-100)
Minimal integration between workforce development initiatives	75.38 (10-100)
Training alone, without additional support to enhance practice	75.38 (40-100)
Lack of motivation to measure progress towards change	72.50 (10-100)
Emphasis of organization on procedural documentation	72.50 (10-100)
Limitations in referral criteria and inclusion criteria for support	64.17 (50-100)
A lack of available specialists or services	49.23 (10-80)

Highly rated common barriers to change included issues within ‘organisational structures’, a lack of ‘collaborative practice’, the ‘organisational culture’ and limited focus on effective ‘implementation strategies’ and ‘sustained’ practice change.

Curiously, ‘a lack of available specialists or services’ was rated as the least important barrier to system change in care provided to families. Similarly, ‘restrictions in referrals of support for families’ as a barrier was also lowly rated and both were not deemed as reaching a consensus level of importance with informants.

#### Strategies: early and/or later in the change process

**Figure 1:**
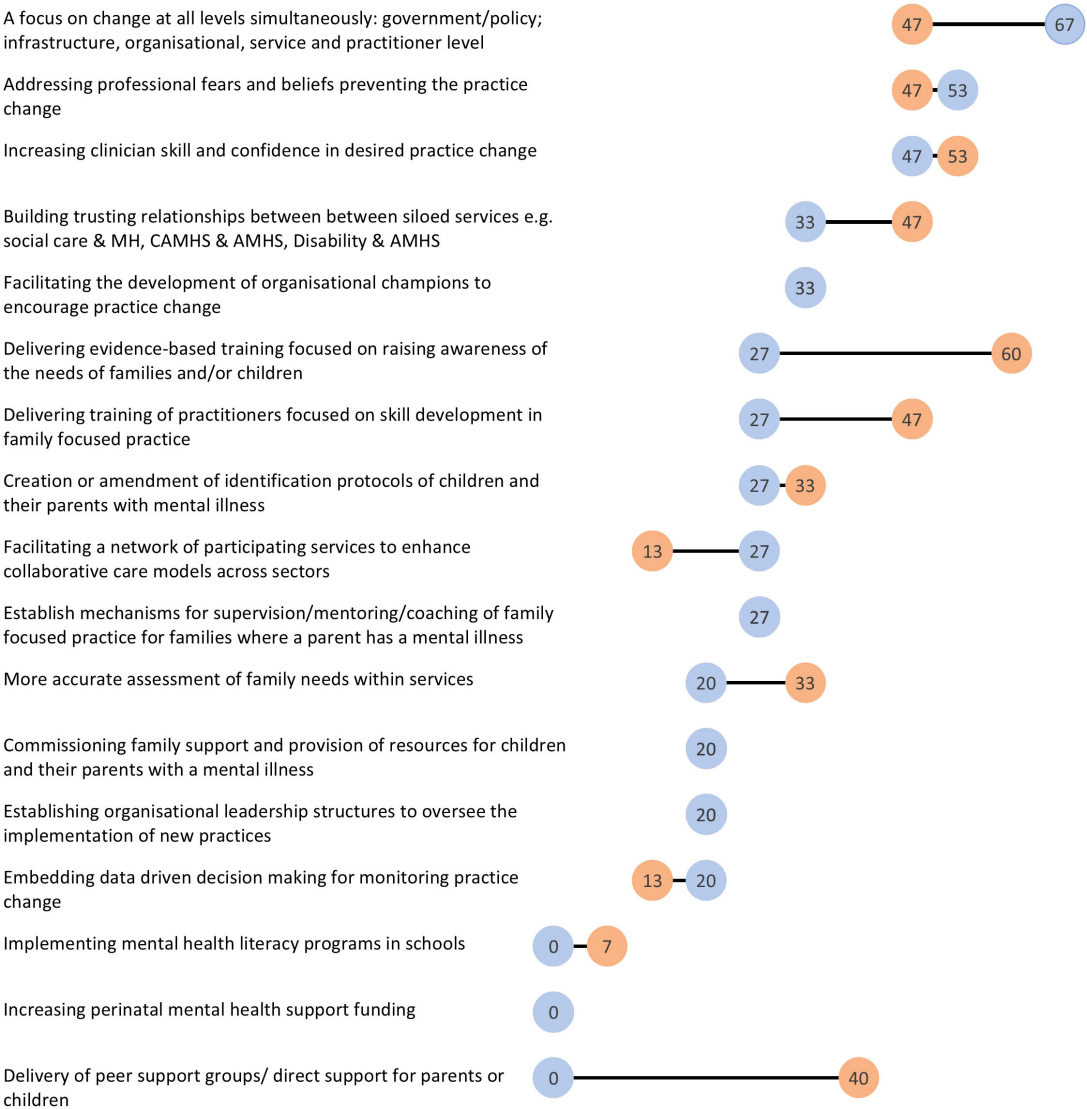
Percentage (%) of respondents (n=18) utilizing strategies Early in the change journey vs for Sustainment of system change. MH, Mental Health; CAMHS, Child and Adolescent Mental Health Service; AMHS, Adult Mental Health.

The first round results indicated that in strategies described by respondents there was an associated journey or an association with time (58). This led to a curiosity as to whether strategies utilized at the earlier stages of the change journey were comparatively different with later stages of the change journey, where there might be more of a focus sustaining practice change. To address this, we asked informants to rate the strategies identified in round one according to what was successfully focused on early in the change process or later in the change process. Additionally, informants were asked to rate the importance out of 100 of the activity focused on early in the change process. Informant ratings are noted in **Table 3** below and the changes in strategies utilized early in the journey and for sustainment are also depicted in **Figure 1** below.

**Table 3:** Respondent ratings of strategies utilized early on in the system change journey compared with later on for sustainment of system change.

Strategy	Utilised EARLY in journey (n, %)	Importance of strategy (EARLY) Mean (range)	Utilised for SUSTAINMENT (n, %)
Establishing organizational leadership structures to oversee the implementation of new practices	3 (20)	96.00 (90-100)	3 (20)
Facilitating a network of participating services to enhance collaborative care models across sectors	2 (13)	94.50 (89-100)	4 (27)
Creation or amendment of identification protocols of children and their parents with mental illness	5 (33)	93.80 (85-100)	4 (27)
Delivering evidence-based training focused on raising awareness of the needs of families and/or children	9 (60)	91.75 (25-100)	4 (27)
Facilitating the development of organizational champions to encourage practice change	5 (33)	91.40 (63-100)	5 (33)
Building trusting relationships between siloed services e.g. social care and MH, CAMHS and Adult Mental Health, disability and Adult Mental health	7 (47)	89.86 (43-100)	5 (33)
A focus on change at all levels simultaneously: government/policy; infrastructure, organizational, service and practitioner level	7 (47)	87.33 (44-100)	10 (67)
Increasing clinician skill and confidence in desired practice change	8 (53)	84.13 (71-100)	7 (47)
Addressing professional fears and beliefs preventing the practice change	7 (47)	79.00 (50-100)	8 (53)
More accurate assessment of family needs within services	5 (33)	78.17 (14-100)	3 (20)
Establish mechanisms for supervision/mentoring/coaching of FFP for families where a parent has a mental illness	4 (27)	75.25 (61-86)	4 (27)
Commissioning family support and provision of resources for children and their parents with a mental illness	3 (20)	70.00 (25-95)	3 (20)
Delivery of peer support groups/direct support for parents or children	6 (40)	69.40 (54-81)	0
Delivering training of practitioners focused on skill development in FFP	7 (47)	67.50 (25-100)	4 (27)
Implementing mental health literacy programs in schools	1 (7)	53.00 (53)	0
Embedding data driven decision making for monitoring practice change	2 (13)	39.00 (25-53)	3 (20)
Increasing perinatal mental health support funding	0	0.00	0

More commonly used strategies *early in the system change journey* included a focus on areas such as ‘awareness raising training’, addressing ‘skills and confidence’ and ‘belief systems’ of practitioners. ‘Building trusting relationships to break down silos’ and ‘increasing collaborative care’ were also highly rated and utilized at the beginning of the change process within a country.

A rating of importance was also included for strategies that informants used early on, and is noted in the middle column of **Table 3**. Highest ratings of importance included ‘establishing leadership structures to oversee implementation’, ‘facilitating a network to enhance collaborative care across sectors’, ‘creation or amendment of identification protocols of children and parents’, ‘delivering evidence-based training to raise awareness of families’ and the development of ‘organisational champions in the practice change’. These results suggest a degree of focus on various organizational levers were seen as important to concentrate on early, as well as a focus on establishing networks to support collaborative care. For the informants who selected these strategies, they were rated as highly important in the change journey.

**Figure 1** demonstrates the shifts in what informants utilized early in the change journey compared with sustaining system change. Commissioning ‘perinatal mental health support’ or ‘mental health literacy programs in schools’ were least likely to be used as a change strategy at the beginning of a change journey and were not utilized at all during sustainment. Offering support directly to families and/or children was more commonly done early in the journey but only to a limited extent. Likewise, data driven decision making was minimally used as a strategy on its own.

A focus on multi-level system change simultaneously at the ‘government/policy, organizational and practitioner level’ was a focus both early on in the journey and also for sustaining change. When examining strategies important for sustainment, this focus on multi-level change was the most commonly rated strategy, more so than early on in the journey. This indicates that informants highlighted the use of this strategy throughout, but it was more of a dedicated focus in the sustainment period.

Other highly utilized strategies in the sustainment phase were a focus on addressing ‘professional belief systems’ and enhancing ‘professional skill and confidence’. Collaborative care and building trusting relationships between siloed services (e.g. adult and child mental health services) was important at the beginning and remained high during sustainment. Lowest utilized strategies included ‘perinatal mental health funding’, ‘mental health literacy in schools’ and ‘delivering peer support programs and support for parents and children’.

## Discussion

The occurrence of mental illness across generations is identified as a ‘wicked’ problem (65), that requires a shift within a complex service system towards an early intervention and prevention response to promote wellbeing of parents and their children (42). This Delphi study examined systems change experts’ opinions on the crucial aspects of system change internationally to enhance the care provided to families where a parent has a mental illness. While there were significantly fewer participants in the second round, the study results indicate that a focus on systems change supporting families where a parent has a mental illness appears to involve the combination of many strategies and factors targeting policy, organizational and practitioner change. Consensus was reached on a number of mechanisms and levers for seeking change that had worked across a number of contexts and countries. Informants reported multiple different levers of workforce development strategies and were at different stages of the change continuum (58); and the success of their target strategy differed, and was related to the context and stage of the change journey underpinning the initiative.

Overall, country-specific system-change experts (informants) nominated multiple targeted strategies they felt were important for system change within their respective countries. Some commonalities were found across countries in identifying *what has worked* to change the system(s) of care provided to families where a parent has a mental illness. The study particularly focused on success strategies that had worked (according to the informant) within their respective country.

At the level of the practitioner, respondents nominated strategies such as awareness raising of the issues faced by families, as well as targeted training to improve practice competency, either in discrete family interventions or in more general family focused practice competencies. For organizational change, informants highlighted structural alignment at leadership levels to ensure buy-in, alongside organizational protocols and data systems that reinforced and monitored the desired practice with families. At the system level, informants highlighted the establishment of collaborative networks across service systems such as child and adult mental health and social care, sector reform, legislation that targeted care requirements, and practices that helped to address family needs such as supporting parenting in adult mental health. Overall, however, the most common successful strategy selected was targeting multiple drivers of change simultaneously to facilitate a multi-level directive for the desired change (“*A focus on change at all levels simultaneously: government/policy; infrastructure, organizational, service and practitioner level”).* This highly rated target suggests in this study that creating a favourable policy and organizational environment that supports a new or enhanced way of providing care to families - simultaneously with the service and practitioner delivering or implementing this new form of practice - is one of the most successful approaches to improving the system of care for families where a parent has a mental illness, according to our informants.

Expanding on the strategies highlighted at the practitioner level, there was a strong focus on initiatives to build the competency of the workforce in the desired practice. Our study also sought to understand whether targets for change differed depending on whether they focused early on in the change journey of a new initiative or later on when efforts were more focused on sustaining the initiative’s outcomes. Early on in a system change initiative, several components of building workforce competency were rated as important to success. These were delivering awareness raising training to practitioners on the needs of families, training in the skills needed to deliver FFP and the development of mentoring and supervision mechanisms in FFP. For more sustained competency building however, informants focused on the need to have an enduring focus on enhancing the skills and confidence of practitioners in the desired practice change and a need to continue to challenge belief systems and fears that might get in the way of embedding the practice change in their way of working.

The results suggest that a scaffolded process to building workforce capacity to change or expanding the scope of their practice may be needed in this field of practice. Likewise, while awareness raising and skills training have previously been found to be necessary components of building family-focused practice in practitioners working with parents and/or children, they have been found to be inadequate on their own (35, 47, 66, 67). Practice change requires the practitioner to integrate the new knowledge they gain through their practice, with support to navigate the hurdles and challenges inherent in their real-world setting (47, 67). Continued practice also requires ongoing attention to competence building in the light of a practitioner’s somewhat fluctuating access to utilize their new skills if they have a mixed client base or dealing with multiple presenting issues from clients (35, 48). The use of regular supervision might also be important here in supporting workforce competency, as it can provide opportunities for continued practice and an avenue to address some of the barriers to implementing the skills in the ‘real world’ setting.

Alongside building practitioner competence, organizational level strategies to support practice within the work setting are commonly considered essential to change the care provided to families (1, 28). Of the 10 strategies rated as most important in *what worked*, seven were organizational level strategies related to leadership and their role in endorsing and authorizing change, building accountability systems and integrating shifts in practice into service models. The importance of different roles of leadership in creating an authorising environment to anchor a practice change in FFP is also noted by Allchin, Goodyear (68). They found that senior leadership were seen to be important in setting the authorizing environment for FFP while middle management were important in helping to fit the practice in the everyday work setting (68).

Other organizational level system change initiatives highlighted as important by informants included a focus on an organization’s protocols for the identification of children and their families within the services supporting family members, and embedding assessments of family needs into the workflow of a practitioner. Improving identification and assessment of parents and their children and family is a common focus of many initiatives in this field (13, 19, 42, 69, 70). Mandates for identification are in place in countries like Norway (71), the Netherlands (72) and in parts of Australia (39). However, the success of the translation to practice of these mandated practices is patchy (69, 73). While important, there is growing discussion on how different approaches to identification and assessment can impact families. A study of a mandatory routine identification process in the Netherlands highlighted how identification and assessment through an ‘assessing for risk’ lens can lead to a *truth finding* rather than *an offering of support* outcome (72). As a result, this mandatory process did not actually lead to children getting the support they needed because the tool tended to direct practitioners’ attention away from the child’s needs and more towards reporting to child abuse authorities (72).

The Austrian “It takes a village” study focused on embedding routine identification into initial intake procedure when a parent is entering the adult mental health ward or seeking mental health treatment (13, 46). This targeted change was the result of an extensive codesign process with parents and service providers, whereby it became evident that any identification procedure needed to address the potential hesitancy that many parents with a mental illness have in talking with mental health professional about their child. Many parents with a mental illness are at first hesitant to open up about their child for fear of negative consequences and often benefit from some reassurance of a supportive partnership they have with their health professional (5, 74–77). Practitioners were trained to use a structured tool that included trauma-informed supportive questions during intake at adult mental health service or primary health clinics as the parent sought treatment for their mental health issues. This was followed by a warm referral to a “village facilitator” role in the community to explore the needs of the family in partnership with the parent and also their child(ren) in a strengths-based manner, and using key engagement strategies (13, 46, 75). A key foci showing promise for improving outcomes for families identified in the adult mental health system is in supporting the self-determination of parents and their children as part of the identification process, particularly in cases where families may have had negative and less autonomous interactions when seeking treatment support (5, 34, 74, 78, 79).

System level strategies support practitioner and organizational level initiatives by helping to create or enhance the favorable conditions for change. These were reflected in the results of this study as both barriers and in *what had worked.* System level strategies such as mandates and policies are known drivers for change (27, 28), but they have a long lead time and are only effective when paired with strategies to implement at the organizational level. The lack of a favorable policy environment to mandate change is often seen as a barrier, however, in countries where such legislation exists, it is noted that legislation was only part of the story of change. In Norway, legislative change has provided a catalyst for slow movement towards the translation to practice change across a range of settings. The law regarding children as next of kin (2010) came with a requirement for health institutions/hospitals to appoint child responsible personnel (CRP) to promote and coordinate support to patients in their parental role, and their children (73) and for health professionals across sectors to identify and provide support to patients’ children and families (71). Over 10 years there has been a 54% increase in the CRP positions (80, 81). Paired with localized work in municipalities during 2007–2015 to support young children of parents with mental health, and/or substance abuse problems, the law and these positions have strengthened collaborations and training across service sectors (82).

Without the reinforcement of multiple other active strategies that require a level of accountability within services, e.g., Reporting systems, legislation mandates might not lead to new procedures and practice change. In one region in Norway, an 11-year follow-up on of the use a suite of identification and support interventions revealed that registration of patient’s children had increased, but provision of support to the children was less than 30% (40, 83, 84). Similarly in a region of Australia, a change in the mental health act that mandated clinicians to recognize and support consumers’ children was not adequately resourced within organizations and consequently did not lead to a change in practice (39). The informants in this study would appear to agree that while mandating strategies like policy and legislation are important and have been helpful, they need to be combined with other organizational level strategies that build local authority along with monitoring and accountability for adherence.

It was surprising to see that providing a direct service to family and/or their children (e.g. peer support programs, family support) was one of the areas identified as important in round one, but rated low in terms of importance in round two. The research field in families where a parent has a mental illness consists primarily of studies that examine the effectiveness of either parenting programs or children’s programs for families where a parent has a mental illness (12, 14, 15, 79, 85–88). Several countries have reported decades of implementing supportive programs for families, but in countries such as the Netherlands, there has been a lack of success in sustainment over time due to a reliance on non-recurrent government funding for the delivery of these programs (20). Sustainment of children’s peer support programs in the case of one country (Australia) has been cemented recently through the establishment of on-going regional funding in response to a Royal Commission into the Mental Health System and an identified need for care for children in these families (89). While on-going government funding may be crucial for practice initiatives that can deliver particular outcomes for families where a parent has a mental illness, it must also be noted here that a focus on implementing family interventions may work in some contexts or countries where there is limited alternative or complementary infrastructure supports such as policy or organizational leadership for a more widespread practitioner and service level change. This may be particularly the case salient for lower income countries or those countries working to raise awareness of the needs for system change to support the unmet needs of these families (20). Outcome studies of low cost and regionally specific family interventions can be an important step in highlighting to policy makers the benefit of future investment and widespread scale up of training and organizational support, for instance, particularly in low resource settings (90, 91). More research is particularly needed in this space with a larger sample size and a more widespread sample of participating countries to further tease this out.

The discrepancy between round one nominations and round two ratings was also seen for *improving mental health literacy in schools* and *increasing the funding for perinatal mental health programs.* In round two, these strategies were only very minimally considered as important or utilized, if at all. Again, this might be a reflection of the smaller sample size in round two. Another potential reason for this could be the perceived limitations of these strategies to influence a wider system of care. It might be the case that informants believed that targeting only one part of the multiple service systems involved in caring for these families (23), is not enough or a good use of resources to shift the system of care to better address the breadth of needs that families may present with. Given the scope of system change these informants were considering, perhaps these strategies were also seen as too narrow for the targeted focus of an initiative to change practice and care models.

A complex area of focus for system change highlighted by informants was addressing the siloed nature of service systems working with these families and promoting collaborative care. Informants rated these as important both early in the change process and for sustainment. The promotion of collaborative care in this field however, particularly between adult and child services, remains a major challenge (29, 92). Family focused practice initiatives in mental health services have included workforce development approaches that include joint cross-sector staff training opportunities to help address a lack of common language or point of foci and understanding that can help promote collaborative care across different sectors including adult and child mental health, education and social care or welfare services (67). However, these and other workforce development initiatives that bring together staff from different sectors still require a supportive organizational environment for translation to practice (67). Another initiative targeted leadership and entailed the creation of collaborative agreements that depicted a collaborative service response between different services supporting families where a parent has a mental illness. These agreements were a codesigned inter-agency protocol that stipulated effective ways of working between services. At the one-year follow-up, promising results were seen for increases in collaborative practice and communication between agencies, however, the initiative’s success was limited by on-going systemic barriers such as poor communication channels, differing interpretations of confidentiality requirements, resource inadequacies, and time and workload issues (93).

## Limitations

Informants were asked to reflect on system change initiatives in respective countries of origin where they had been working and studying system change. They were also expected to provide detail and evaluate initiatives that may have been operating at different levels of system change. It is recognized that their perspective is reliant on the extent of their knowledge and awareness of the activities within their country and the results need to be interpreted with epistemic humility. For example, some participants may have greater knowledge of initiatives at the national and regional level, but know less about discrete local initiatives. While this may limit the breadth of knowledge from the participants about small initiatives that might have been working locally, it is more likely that participants were aware of large-scale initiatives that seek national impact and sustainability. This knowledge, however, is preferable given an understanding of large system change initiatives may contribute more usefully to addressing the knowledge gap around sustainable strategies for a global and strategic audience.

It should also be acknowledged that there was a significant participant drop-off from the first to the second round of the Delphi Survey. This is likely due to the time lapse between the first and second round where a number of respondents changed their work roles or were not available to participate in the second round during the timeframe. A thorough analysis was conducted of the first round data in the timeframe which resulted in another paper using a narrative analysis of the data to understand more of the contextual factors at play in each of the represented countries (58). The design of this study was also highly collaborative and all stages of the survey design, analysis and write-up were conducted by researchers from an international research collaborative for families where a parent had a mental illness (listed co-authors of this paper). While the sample size was small in the second round, the findings do correspond with a number of other implementation science studies in the field (48, 84). We recommend that further research is conducted to confirm the representativeness of the identified factors rated by consensus with a larger sample size across more diverse settings, including non-OECD countries.

This study was specifically designed to address a gap in the field of family mental health research by examining common factors regarding the implementation supports needed to change systems of care that have previously been shown to be successful in various countries internationally. The study was conducted by a team of international researchers who focus on system change for the Prato Research Collaborative on child, parent and family mental health and builds on previous research on system change initiatives, examined across different contexts (20). Recruitment for the Delphi Study involved snowball sampling from the existing network of the Prato Collaborative researchers. Through this method, we were able to seek input from 17 countries in round one and 9 countries in round two. The countries represented are all middle to high income with well-established health and social care sectors. It must be noted that while consensus was reached across the aforementioned countries (as defined by the Delphi methodology), we cannot make strong claims for generalizability to countries where specific contextual factors (such as culture and wealth) require unique approaches to promulgating change. We therefore recommend that further research is required to confirm the representativeness of the identified factors rated by consensus with a larger sample size across more diverse settings, including non-OECD countries. We also recommend that each of these factors identified need to be considered following a planning and implementation process whereby the needs of the setting are identified and taken into account. It might be useful here to consider the use of two well used implementation science frameworks, namely the EPIS (exploration, preparation, implementation, sustainment) framework (94) and/or the CFIR (the Consolidated Framework for Implementation Research) framework (27). Each of these frameworks can help create structural guidance to examining specific barriers and enablers and help set out targets for the implementation of interventions and system change initiatives (95, 96).

## Implications and conclusions

System change is a sought after target when aiming to improve functioning within a system or to respond to and better serve community needs (41). Typically though, a focus on system change is ambitious and wide ranging in impact (97), it can require decades to understand how the venture leads to better outcomes for the population (98). Our informants identified multi-systemic and complex strategies as drivers that *have worked* to change systems of care in this field. Rarely did informants highlight a narrow focus like perinatal care or mental health promotion as a successful strategy, except in focusing on mono-level strategies early on in the change journey.

System change outcomes are often emergent and a non-linear response to the interaction of multiple elements and drivers. This may include the culmination of multiple elements over time that result in a tipping point that drives and builds the directive for system change. It can be difficult to pinpoint any one strategy, which makes measurement of success even more challenging (98). Consequently there is a dearth of understanding regarding key processes of systems change and how they interact to improve systems of care (41). Taking an approach that seeks the opinions of informants immersed in studying or understanding the factors that were important for system change is one important way to address a lack of knowledge in this area.

Success in system change requires a focus on change at all levels of the system simultaneously as evidenced in this Delphi study. While small wins might be important early on for momentum building, bigger impact requires the interconnection of multiple drivers such as leadership support, the use of relevant data and reporting mechanisms, establishing practice competency and collaborative care, and being able to reflect and adapt to changing conditions and structural barriers. This interconnection between drivers is a much-needed area of development for future research. A focus on integrated systems of care for families where a parent has a mental illness is the ultimate goal, where all sectors and professionals are collaborating and are equipped to meet the emerging mental health challenges of these families and especially their children, helping to address this ‘wicked problem’ of the intergenerational impacts of mental illness.

## Data Availability

The original contributions presented in the study are publicly available. The de-identified datasets used in this study can be found at https://doi.org/10.26180/27157746 and in the **Supplementary Material**.

## Supplementary material

Supplementary Table 1 Table of participant demographics from Round 1 and Round 2 of the Delphi Study on System Change.

Supplementary Table 2 System Change Definitions (round two).

Supplementary Table 3 Requirements for system change definition.
