# Supplementary material for: International perspectives on implementation of system change in family mental health
Source: Front Psychiatry. 2026 Feb 24;17:1705868. doi: 10.3389/fpsyt.2026.1705868 (PMC12971957; doi:10.3389/fpsyt.2026.1705868)
Supplement: Supplementary Table 1 — Table of participant demographics from Round 1 and Round 2 of the Delphi Study on System Change. [file Table1.docx]

**S1 Table.** **Table of participant demographics from Round 1 and Round 2 of the Delphi Study on System Change**

| Country | Round 1  N=91  n (%) | Round 2  N=15  n (%) |
| --- | --- | --- |
| Australia | 7 (8) | 3 (20) |
| Canada | 3 (4) | 1 (7) |
| Denmark | 6 (7) | 2 (13) |
| England, UK | 9 (10) | 1 (7) |
| Finland | 10 (11) | 0 |
| Iceland | 1 (1) | 1 (7) |
| Ireland | 11 (12) | 1 (7) |
| Italy | 1 (1) | 0 |
| Israel | 4 (4) | 0 |
| Netherlands | 2 (3) | 0 |
| New Zealand | 6 (7) | 1 (7) |
| Northern Ireland | 4 (4) | 3 (20) |
| Norway | 9 (10) | 1 (7) |
| Serbia | 7 (8) | 0 |
| Sweden | 6 (7) | 0 |
| USA | 4 (4) | 0 |
| UAE | 1 (1) | 0 |
| Age | n (%) | n (%) |
| Unknown | 2 (2) | 0 |
| 20-29 | 2 (2) | 0 |
| 30-39 | 11 (12) | 2 (13) |
| 40-49 | 28 (31) | 4 (27) |
| 50-59 | 36 (40) | 6 (40) |
| 60-69 | 12 (13) | 2 (13) |
| 70+ | 0 | 1 (7) |
| Gender | n (%) | n (%) |
| Male | 24 (26) | 3 (20) |
| Female | 64 (74) | 12 (80) |
| Years of experience | n (%) | n (%) |
| 1-5 | 3 (3) | 1 (7) |
| 6-10 | 12 (14) | 0 |
| >10 | 76 (84) | 14 (93) |
| Current Professional Role | n (%) | n (%) |
| Clinician | 29 (32) | 0 |
| Government advisor | 4 (4) | 2 (13) |
| Program Manager/Service director | 20 (22) | 1 (7) |
| Project manager | 5 (5) | 3 (20) |
| Research/Academic | 12 (13) | 5 (33) |
| Workforce trainer/ advisor | 15 (16) | 2 (13) |
| Other | 6 (7) | 2 (13) |
| Professional Background | n (%) | n (%) |
| Counsellor/Child & Family Welfare | 5 (5) | 0 |
| Health Education | 1 (1) | 0 |
| Leadership Management | 3 (3) | 0 |
| Medicine | 2 (3) | 0 |
| MH consumer | 1 (1) | 0 |
| Nurse | 18 (20) | 4 (27) |
| Occupational Therapist | 5 (5) | 1 (7) |
| Psychiatrist | 11 (12) | 1 (7) |
| Psychologist | 8 (9) | 1 (7) |
| Social Work | 31 (34) | 7 (47) |
| Sociology | 2 (2) | 1 (7) |
| other | 4 (4) | 0 |
| Type of organization | n (%) | n (%) |
| Addiction Health Service | 2 (2) | 0 |
| Child/ Adolescent Mental Health | 5 (5) | 0 |
| Child Welfare | 2 (2) | 1 (7) |
| Community Health | 3 (3) | 0 |
| Government agency | 7 (8) | 0 |
| Hospital | 6 (7) | 1 (7) |
| Non-Government Organization | 10 (11) | 0 |
| Private hospital/clinic | 4 (4) | 0 |
| Public Mental Health Service | 31 (34) | 6 (40) |
| University | 11 (12) | 5 (33) |
| Workforce or training organization | 6 (7) | 1 (7) |
| Other | 4 (4) | 1 (7) |
