# Supplementary material for: International perspectives on implementation of system change in family mental health
Source: Front Psychiatry. 2026 Feb 24;17:1705868. doi: 10.3389/fpsyt.2026.1705868 (PMC12971957; doi:10.3389/fpsyt.2026.1705868)
Supplement: Supplementary Table 2 — System Change Definitions (round two). [file Table2.docx]

**S2 Table. System Change Definitions (round two).**

Comments provided by participants in reviewing the definition of system change, and how the definition was edited in response.

| Definition 1 Comments: | How we edited the definition |
| --- | --- |
| This may be unnecessarily pedantic - to me systems change suggests a process which is fairly comprehensive. From this definition, and I may have misinterpreted it, relatively minor change in any of the areas identified would be viewed as systems change. It could also be positive or negative change but that's maybe deliberate. Maybe if it was 'to more effectively identify and support' that would clarify it? | Added the term ‘*collectively contributes’* to highlight the need for multiple strategies working together.  Redefined the desired effect as  *‘improving outcomes for parents with mental illness, their children and their families’* to emphasise the need for strategies to be effective at changing outcomes |
| I would include ........any adult or children's workforce ..... | Have retained ‘any workforce’ to enable the definition to be inclusive beyond adult and children i.e. family, government, |
| The word "any" disturbs me. System change has to be built on Evidence Based support for these families and implemented systematically with EB procedures. But it is important to acknowledge in policy and legislation that parents with mental illness need support. | While retained ‘any’, redefined an outcome as *‘improving outcomes for parents with mental illness, their children and their families’* to more clearly define the strategies as effective for the population |
| Although I am rating systems change as 9 in importance I don't actually agree with the definition provided. Any of those actions in and of themselves do not constitute systems change. They may make a significant contribution or provide a model or example of 'better' practice however this does not change a system. Herein lies the problem | Added the term ‘*collectively contributes’* to highlight the need for multiple strategies to change systems |
| System change needs to include the voice of the child and parent and how their experience the change and support. | Redefined ‘identify and support parents with mental illness and their families, including their children’ to ‘improving outcomes for parents with mental illness, their children and their families’ to shift the focus on what is done for/to them to improvements that are important to them |
| I would include reference to education. Interdisciplinary education in this area very important at undergrad and post grad level and in service training also very important. | This would be included in the broad context of workforce strategy |
| The term "other health promotion strategy" is a little broad. Some strategies are not evidence based and/or are aimed at a population group that is not particularly affected by the issue. | Defined an outcome as *‘improving outcomes for parents with mental illness, their children and their families’* to more clearly define the strategies as effective for the population |
| I think system level change requires much more than promotion as a single strategy | Added the term ‘*collectively contributes’* to highlight the need for multiple strategies |
