# Supplementary material for: International perspectives on implementation of system change in family mental health
Source: Front Psychiatry. 2026 Feb 24;17:1705868. doi: 10.3389/fpsyt.2026.1705868 (PMC12971957; doi:10.3389/fpsyt.2026.1705868)
Supplement: Supplementary Table 3 — Requirements for system change definition. [file Table3.docx]

**S3 Table.** **Requirements for system change definition.**

Comments provided by participants in reviewing the definition of what system change requires, and how the definition was edited in response.

| Definition 2 Comments | How we addressed them: |
| --- | --- |
| Yes, I think the role of wider social policy issues - especially around poverty and inequities - are of central importance. | The statement has been strengthened by the inclusion of health policy : ‘*public health, social and health policy development’* |
| System approach like the Solihull Approach in practice is needed. | The Solihull Approach is an example of regional cross sectorial partnerships around a common goal |
| could start with families.... then think about how and what needs to connect | The statement has been strengthened to include families in the partners list |
| Two comments - a) your rating scale should be from 0-10  b) you've missed child welfare" | Welfare in its broadest context has been understood as including child welfare |
| Which include government recognition and support | The statement has been strengthened by the inclusion of health policy : ‘*public health, social and health policy development’* |
| I would include voluntary sector also as they work closely with statutory services in supporting families. | In each of these broad areas there are public, private and voluntary agencies who engage with families |
| To be most effective in terms of impact on the system this partnership work is critical. However there are some internal issues that may require addressing to get parts of the system 'ready' for the change. | A system’s internal readiness for change and the mismatch of fit between systems is the work of partnerships and collaborations. The statement has been strengthened by adding ‘*collaborations’* to indicate the ongoing nature of the work |
| Systemic change can only be maintained where there are strong partnerships across services and sectors | ‘*collaborations’* has been added to strengthen the statement, as well as ‘*services and sectors’* |
| Maybe add housing | Agreed that housing security is vital for family wellbeing. Welfare and social policy in their broadest context have been understood as including Housing |
